# The Transcriptional Profiling of Glycogenes Associated with Hepatocellular Carcinoma Metastasis

**DOI:** 10.1371/journal.pone.0107941

**Published:** 2014-09-18

**Authors:** Tianhua Liu, Shu Zhang, Jie Chen, Kai Jiang, Qinle Zhang, Kun Guo, Yinkun Liu

**Affiliations:** 1 Liver Cancer Institute, Zhongshan Hospital, Fudan University, Shanghai, People’s Republic of China; 2 Cancer Research Center, Institute of Biomedical Science, Fudan University, Shanghai, People’s Republic of China; The University of Hong Kong, China

## Abstract

**Background and objective:**

Metastasis is one of the important reasons for the poor prognosis of hepatocellular carcinoma (HCC), abnormal glycosylation plays a pivotal role in HCC metastasis. The goal of this study was to screen and validate the transcriptional profiling of glycogenes associated with HCC metastasis.

**Methodology:**

The differentially transcribed glycogenes were screened out by the Human Glycosylation RT^2^ Profiler PCR Array, and were identified by qRT-PCR in human HCC cell lines and their orthotopic xenograft tumors. Further analyses were performed with *K-mean* clustering, Gene Ontology (GO) and ingenuity pathways analysis (IPA). Four differentially transcribed glycogenes were validated in clinical cancer specimens by qRT-PCR.

**Results:**

A total of thirty-three differentially transcribed glycogenes were obtained by comparison the transcription in the metastatic human HCC cell lines (MHCC97L, MHCC97H and HCCLM3) with the transcription in the non-metastatic HCC cell line Hep3B. Seven differentially transcribed glycogenes were selected to further identification in human HCC cell lines and their orthotopic xenograft tumors. According to their trends by *K*-mean clustering, all of the differentially transcribed glycogenes were classified in six clusters. GO analysis of the differentially transcribed glycogenes described them in biological process, subcellular location and molecular function. Furthermore, the partial regulatory network of the differentially transcribed glycogenes was acquired through the IPA. The transcription levels of *galnt3*, *gcnt3*, *man1a1*, *mgat5b* in non-metastatic and metastatic HCC clinical cancer specimens showed the same changing trends with the results in human HCC cell lines and their orthotopic xenograft tumors, and the divergent transcription levels of *gcnt3* and *mgat5b* were statistically significant.

**Conclusions:**

The transcriptional profiling of glycogenes associated with HCC metastasis was obtained and validated in this study and it might provide novel drug targets and potential biological markers for HCC metastasis.

## Introduction

Hepatocellular carcinoma (HCC), which is the sixth common neoplasm, ranks the third in cancer mortality in the world [1]. 80% of the HCC are mainly found in eastern Asia and sub-Saharan Africa [2]. HCC is a complex process mediated by multiple genes. The risk factors for the development of HCC, which interact and cooperate with each other, could increase the probability of HCC tumorigenesis. Most people suffering from HCC will die within one year after its detection [3]. One reason for the high mortality can in part be attributed to extrahepatic metastasis [4] and the bottleneck in treatment of HCC is to prevent extrahepatic metastasis. The understanding of the gene transcription profiling underlying HCC metastasis will provide us new theoretical basis for HCC diagnosis and treatment.

Glycosylation, which can be found in a variety of physiological and pathological events, is one of the most important kinds of posttranslational modifications. More than 50% of proteins in nature are presumed to have undergone glycosylation [5]. These glycans not only alter the structures and functions of glycoproteins, but also are crucial for cell adhesion and cellular signal transduction. Other than that, aberrant glycosylation also plays a key role in the underlying mechanism of a variety of diseases [6]. Glycans are formed by the catalytic activity of enzymes such as glycosyltransferases and glycosidases. The alterations in transcription and translation levels of enzymes are related to corresponding changes in the glycan branched structures. Currently, studies of the aberrant glycosylation in HCC have been paid much attention and certain achievements have been made. *mgat3* and *mgat5* were proved to be over-expressed in human HCC cell lines, knockdown of *mgat3* could promote cells invasion and increase the resistance to 5-fluorouracil in vitro [7]. However, the silence of *mgat5* in cells could inhibit invasion and increase sensitivity to 5-fluorouracil in vitro. *mgat4a* was vital to tumor migration and metastasis through altering the glycosylation of CD147 [8]. It was also reported that transcription levels of *mgat4b*, and *mgat5* were increased in mice with HCC [9]. Recently, further analysis revealed that *mgat5* could partially decrease cell adhesion and promote cell proliferation through RPTPκ [10].

In this study, we obtained and identified the transcriptional profiling of glycogenes in human HCC cell lines with or without metastasis potential and orthotopic xenograft tumors by PCR Array and qRT-PCR. The differentially transcribed glycogenes were classified and described by *K*-mean clustering and GO analysis, respectively. The possible regulatory network of them was built by the IPA. Furthermore, the differently transcribed glycogenges were validated in clinical cancer specimens by PCR Array. This work might provide the novel potential drug targets and the potential biological markers of HCC.

## Materials and Methods

### Ethics Statement

Access to human tissues complied with both Chinese laws and the guidelines of the Ethics Committee and this study was approved by the Research Ethics Committee of Zhongshan Hospital and First Affiliated Hospital of Guangxi Medical University. All participants have given written informed consent.

### Cell Culture

The Human HCC cell lines with different metastatic potentials (MHCC97L, MHCC97H and HCCLM3) have the same genetic background and were established in our Liver Cancer Institute [11]–[14]. All of them were cultured in Dulbecco’s modified Eagle’s (DMEM) medium (Gibco, USA) supplemented with 10% fetal bovine serum (FBS, Gibco, USA). Hep3B, the non-metastatic HCC cell line (ATCC number HB-8065), was generously provided by Cornell University and was cultured in minimum essential medium (MEM) medium (Gibco, USA) supplemented with 10% FBS (Gibco, USA). All of these cells were incubated at 37°C with a 5% CO_2_ in air atmosphere.

### HCC orthotopic xenograft tumor model in nude mice

The male BALB/C nude mice (5 to 6-week-old) we obtained were from Shanghai Institute of Materia Medica (Chinese Academy of Sciences, Shanghai, China). The orthotopic xenograft tumor models were established as described previously [15]–[17]. Simply, 1×10^7^ Hep3B, MHCC97L, MHCC97H or HCCLM3 cell lines were injected subcutaneously into the upper left flank region of nude mice, respectively. After tumor formation, the tumors were cut into 2 mm×2 mm×2 mm sized pieces. Nude mice that were anaesthetized with pentobarbital 45 mg/kg by intraperitoneal injection after regular disinfection were dissected and then the tumor tissue lumps were implanted into livers of nude mice. The mice were slaughtered at the 35th day after tumor implantation and the orthotopic xenograft tumors were immediately placed in liquid nitrogen. All of the in vivo experiments carried out were strictly complied with the protocol which was approved by the Shanghai Medical Experimental Animal Care Committee (Permit Number: 2009-0082).

### Clinical cancer specimens

Fifteen HCC patients under-going resection in 2012 in Zhongshan Hospital, Fudan University (Shanghai, China) and First Affiliated Hospital of Guangxi Medical University (Nanning, China) were enrolled in this study, including ten patients with non-metastatic HCC and five patients with metastatic HCC. All the tissue samples collected from the patients were performed to qRT-PCR analysis. The detailed information of these fifteen patients was described in Table S1.

### Human Glycosylation PCR Array and qRT-PCR

Total RNA was harvested from cultured cells, orthotopic xenograft tumors and clinical cancer specimens with TRIzol (Invitrogen) and purified with the RNeasy MinElute Cleanup Kit (Qiagen) according to the manufacturer's instruction. After quantification using a Nanodrop spectrophotometer, 10 µg total RNA was reversed transcribed into cDNA using the Revert Aid First Strand cDNA Synthesis Kits (Fermentas), respectively. Besides, cDNA prepared respectively with Super Array PCR master mix (Cat. No. PA-112) was needed in order to perform PCR Array.

The Human Glycosylation RT^2^ Profiler PCR Array, including 84 key genes encoding glycan processing enzymes i.e. glycosyltransferase and glycosidase for several important sugars: galactose, glucose, mannose, N-acetylgalactosamine, N-acetylglucosamine, fucose and sialic acid, was designed and produced by the Qiagen company (German).

The PCR Array and qRT-PCR were performed on IQ5 machine (Bio-Rad) and the PCR cycling condition was set as follows: 95°C for 5 min, 40 cycles of 95°C for 15 s, 60°C for 15 s and 72°C for 20 s. Each test was run three times and the mean was taken to eradicate any discrepancies.

The used primer sequences in qRT-PCR were summarized in Table 1.

**Table 1:** Sequences of qRT-PCR primers.

Glycogene	Primer Sequence
*c1galt1*	F: 5′-ATGAGTGGAGGAGCAGGATA-3′
	R: 5′-TCTGGCACAAAGGGATGAA-3′
*galnt3*	F: 5′-CTGCCTCTCCAGGCAACG-3′
	R: 5′-GTAGTACCTGGCGGGTGGG-3′
*gcnt3*	F: 5′-AGATGTTGAATGGGAGGAATAG-3′
	R: 5′-TGTTGGTTAGGTGTAATGTGTCTC-3′
*man1a1*	F: 5′-CTCTCAGCCTACTATCTGTCTGG-3′
	R: 5′-CAGTTCCTTCCAATACCACTTT-3′
*mgat4a*	F: 5′-GCAACGGAAGAACAGGAGTTTC-3′
	R: 5′-AGGTTGGCTACAACACCATGT-3′
*mgat5*	F: 5′-GCTGCCCCTGTAGGAGAC-3′
	R: 5′-GAATCAAGGACTCGGAGCAT-3′
*mgat5b*	F: 5′-ATGAGTGGAGGAGCAGGATA-3′
	R: 5′-TCTGGCACAAAGGGATGAA-3′
*st3gal1*	F: 5′-CACGAATGGCGTTGGTCTAC-3′
	R: 5′-CTCAATCAAAAGGGATGGCA-3′
*β-Actin*	F: 5′-CATGTACGTTGCTATCCAGGC-3′
	R: 5′-CTCCTTAATGTCACGCACGAT-3′

### Statistical analysis and database searching

Take advantage of the Ct obtained from PCR-Array, the MeV 4.8.1 was used to map the hierarchical clustering of all the glycogenes in the PCR Array, the differentially transcribed glycogenes generated and altered N-linked glycans, O-linked glycans and glycosphingolipids, respectively.

The fold change was normalized to the expression of the housekeeping gene, *β-Actin,* and was calculated respectively for each cell using 2^−ΔΔCt^, where ΔΔCt = (Ct target gene-*β-Actin*) MHCC97L/MHCC97H/HCCLM3-(Ct target gene-*β-Actin*) Hep3B. Glycogene with differences greater than 2-fold (P≤0.05) was considered as the differentially transcribed glycogenes and they were divided into six clusters by a *K-*means approach, which is a kind of clustering algorithm for grouping genes or proteins with a similar expression pattern [17], [18] and the GO was used to describe. IPA (Ingenuity System Inc, USA) is a proof of knowledge based comprehensive software of data analysis that can help researchers model, analyze, and understand the complex biological and chemical systems in life science research [19], [20]. The differentially transcribed glycogenes were uploaded to the database to explore regulatory network. In the analysis of validation in clinical cancer specimens, the housekeeping gene *β-Actin* was as the reference and the relative expressions value were expressed by ΔCt = Ct target gene-*β-Actin*, which means that the higher value of ΔCt of the glycogene, the lower its transcription level.

## Results

### The transcriptional profiling of glycogenes

**Figure 1:**
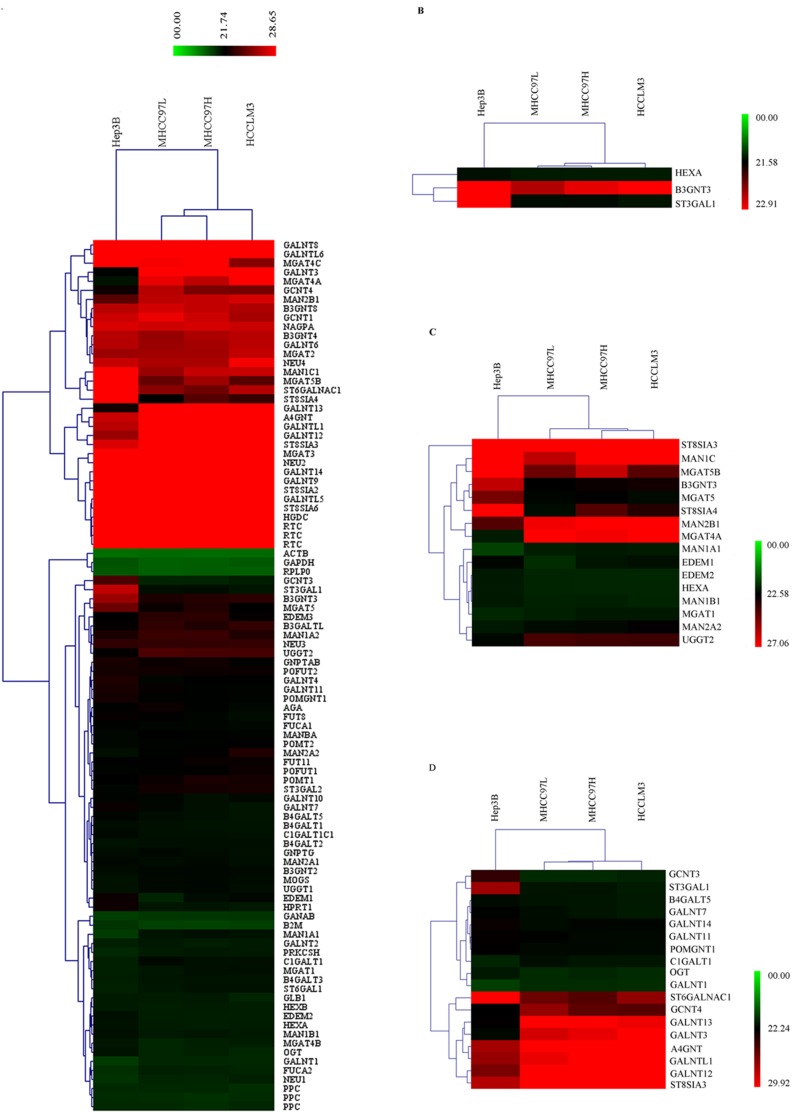
Hierarchical clustering. (A) Hierarchical clustering of 84 glycogenes. Each row represented a single gene; each column represented a cell line. The expression levels of glycogenes, which were the average value of three test results, were shown by the color scale: red represents a target gene with high Ct value while green represents a target gene with low Ct value. (B) Hierarchical clustering of 3 glycogenes acted on glycosphingolipids. (C) Hierarchical clustering of 16 glycogenes generated and altered mature N-linked glycans. (D) Hierarchical clustering of 18 glycogenes generated and altered mature O-linked glycans.

The transcriptional profiling of the 84 glycogenes was obtained by the Human Glycosylation RT^2^ Profiler PCR Array and was used to produce a hierarchical clustering scheme (Figure. 1A). A total of thirty-three glycogenes were differentially transcribed. Among them, sixteen glycogenes generated and altered mature N-linked glycans, eighteen glycogenes were concerned in the synthesis and processing of O-linked glycans, three glycogenes acted on glycosphingolipids (Figure 1B–D). Double counting did occur among four glycogenes *b3gnt3*, *hexa*, *st3gal1*, *st8sia3*, which took part in the synthesis of two kinds of glycans. A total of eighteen mRNA, such as *mgat5*, *st3gal1*, *st8sia4*, were up-regulated in the HCC cell lines with different metastatic potentials (MHCC97L, MHCC97H and HCCLM3) compared with the non-metastatic HCC cell line Hep3B, while fifteen mRNA like *c1galt1*, *gcent4*, *man1a1* were down-regulated (Table 2 and 3). *st3gal1* could mediate the sialylation of T antigen and the increase of *st3gal1* expression had been shown to be one of the major mechanisms responsible for the sialylation of T antigen [21]. Sialyl-T antigen was tumor-associated carbohydrate antigen whose expression was largely increased in some types of cancers and associated with poor prognosis [22]. Polysialic acid was a carbohydrate composed of a linear homopolymer of α2, 8-linked sialic acid residues and was with a large amount of negative charge. The presence of polysialic acid attenuates the adhesive property of neural cell adhesion molecule and increases cellular motility. *st8sia4* was one of the α2, 8-sialyltransferases which could add oligosialic and polysialic acid into various sialylated N-acetyllactosaminyl oligosaccharides [23]–[25]. Amounts of β-1, 6-GlcNAc branched N-Glycan were commonly increased in malignancies and correlated with disease progression. *mgat5* was required in the biosynthesis of β-1, 6-GlcNAc-branched N-linked glycans attaching to cell surface and secreted glycoproteins.

**Table 2:** Up-regulated glycogenes in HCC cell lines with different metastatic potentials.

	GeneSymbol	UniGene ID	GeneBank ID	Functional GeneGrouping	Fold Change(MHCC97L/Hep3B)	Fold Change(MHCC97H/Hep3B)	FoldChange(HCC LM3/Hep3B)
**1**	*b3gnt3*	Hs.657825	NM_014256	N-acetylglucosaminyltransferases	14.32	10.34	10.13
**2**	*b4galt5*	Hs.370487	NM_004776	Galactosyltransferases	2.07	2.25	2.99
**3**	*edem1*	Hs.224616	NM_014674	Mannosidases	14.12	3.05	2.31
**4**	*edem2*	Hs.356273	NM_018217	Mannosidases	2.68	2.48	2.71
**5**	*galnt11*	Hs.647109	NM_022087	N-acetylgalactosaminyltransferases	1.80*	2.14	2.39
**6**	*galnt4*	Hs.25130	NM_003774	N-acetylgalactosaminyltransferases	3.25	2.03	2.16
**7**	*galnt7*	Hs.548088	NM_017423	N-acetylgalactosaminyltransferases	2.38	3.48	5.24
**8**	*gcnt3*	Hs.194710	NM_004751	N-acetylglucosaminyltransferases	47.18	41.36	28.84
**9**	*hexa*	Hs.604479	NM_000520	Galactosides & Glucosidases & Hexosaminidases	2.50	2.36	2.41
**10**	*man1b1*	Hs.279881	NM_016219	Mannosidases	2.27	1.73*	2.33
**11**	*man1c1*	Hs.197043	NM_020379	Mannosidases	30.06	11.55	11.47
**12**	*mgat5*	Hs.651869	NM_002410	N-acetylglucosaminyltransferases	7.94	4.69	9.85
**13**	*mgat5b*	Hs.144531	NM_144677	N-acetylglucosaminyltransferases	240.52	71.51	292.04
**14**	*ogt*	Hs.405410	NM_181673	N-acetylglucosaminyltransferases	3.97	2.51	4.00
**15**	*pomgnt1*	Hs.525134	NM_017739	N-acetylglucosaminyltransferases	2.25	1.92**	2.30
**16**	*st3gal1*	Hs.374257	NM_173344	Sialyltransferases	117.78	91.77	166.57
**17**	*st6galnac1*	Hs.105352	NM_018414	Sialyltransferases	280.14	404.50	119.43
**18**	*st8sia4*	Hs.308628	NM_175052	Sialyltransferases	288.02	42.52	81.01

Note: Fold Change>2, P≤0.5; *: Fold Change<2, P≤0.5; **: Fold Change<2, P≥0.5.

**Table 3:** Down-regulated glycogenes in HCC cell lines with different metastatic potentials.

	GeneSymbol	UniGeneID	GeneBankID	Functional Gene Grouping	Fold Change(MHCC97L/Hep3B)	Fold Change(MHCC97H/Hep3B)	Fold Change(HCCLM3/Hep3B)
**1**	*a4gnt*	Hs.278960	NM_016161	N-acetylglucosaminyltransferases	0.20	0.09	0.17
**2**	*c1galt1*	Hs.592180	NM_020156	Galactosyltransferases	0.33	0.45	0.48
**3**	*galnt1*	Hs.514806	NM_020474	N-acetylgalactosaminyltransferases	0.38	0.36	0.39
**4**	*galnt12*	Hs.47099	NM_024642	N-acetylgalactosaminyltransferases	0.01	0.00	0.01
**5**	*galnt13*	Hs.470277	NM_052917	N-acetylgalactosaminyltransferases	0.00	0.00	0.01
**6**	*galnt3*	Hs.170986	NM_004482	N-acetylgalactosaminyltransferases	0.01	0.01	0.00
**7**	*galntl1*	Hs.21035	NM_020692	N-acetylgalactosaminyltransferases	0.22	0.03	0.04
**8**	*gant4*	Hs.272404	NM_016591	N-acetylglucosaminyltransferases	0.06	0.15	0.18
**9**	*man1a1*	Hs.102788	NM_005907	Mannosidases	0.14	0.09	0.12
**10**	*man2a2*	Hs.116459	NM_006122	Mannosidases	0.63*	0.45	0.27
**11**	*man2b1*	Hs.356769	NM_000528	Mannosidases	0.19	0.15	0.11
**12**	*mgat1*	Hs.519818	NM_002406	N-acetylglucosaminyltransferases	0.85*	0.49	0.50
**13**	*mgat4a*	Hs.177576	NM_012214	N-acetylglucosaminyltransferases	0.01	0.01	0.00
**14**	*st8sia3*	Hs.23172	NM_015879	Sialyltransferases	0.01	0.01	0.01
**15**	*uggt2*	Hs.193226	NM_020121	Glucosyltransferases	0.34	0.33	0.36

Note: Fold Change<0.5, P≤0.5; *Fold Change>0.5, P≤0.5.

### Identification in human HCC cell lines and their orthotopic xenograft tumors by qRT-PCR

**Figure 2:**
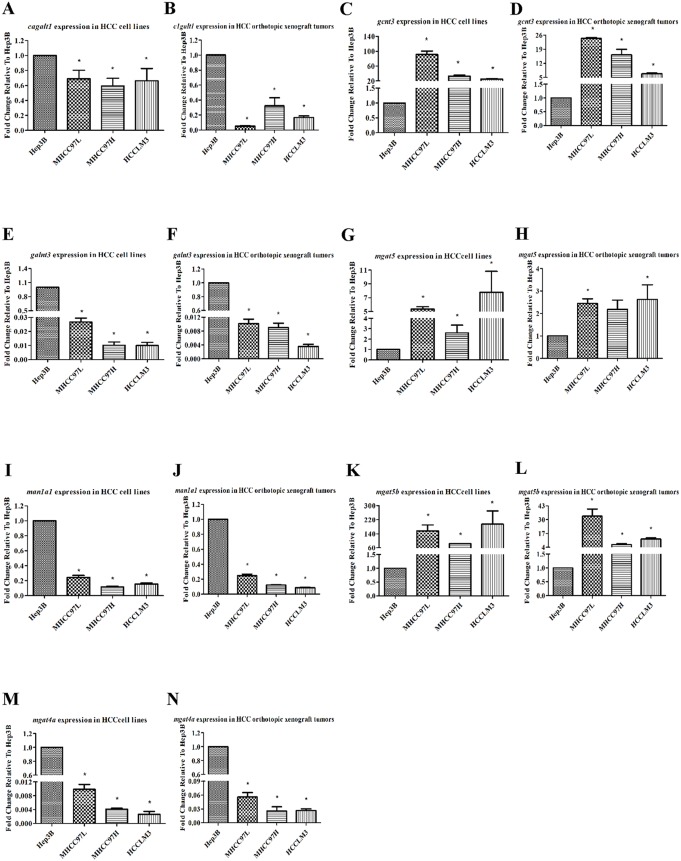
Identification of the differentially transcribed glycogenes in human HCC cell lines and their orthotopic xenograft tumors by qRT-PCR. A, C, E, G, I, K and M were the HCC cell lines with different metastatic potentials (MHCC97L, MHCC97H, HCCLM3) with the comparison to the no-metastatic Hep3B cell line, respectively. B, D, F, H, J, L and N were the orthotopic xenograft tumors of the HCC cell lines MHCC97L, MHCC97H, HCCLM3 comparison to the orthotopic xenograft tumor of the Hep3B cell line. The expression of these glycogenes were normalized against endogenous mRNA of the housekeeping gene, *β-Actin*.

Seven glycogenes: *c1galt1*, *galnt3*, *gcnt3*, *man1a1*, *mgat4a*, *mgat5*, *mgat5b*, were selected to identify the results of the PCR Array by qRT-PCR in human HCC cell lines (Hep3B, MHCC97L, MHCC97H, HCCLM3) and their orthotopic xenograft tumors. The housekeeping gene *β-Actin* was as the reference and normalized the obtained fold changes of differential glycogenes. The results of the human HCC cell lines were shown in Figure 2A, C, E, G, I, K, M, meanwhile the results from the orthotopic xenograft tumors were in Figure 2B, D, F, H, J, L, N. *c1ganlt1*, *man1a1*, *mgat4a*, *galnt3* were all down-regulated whereas *gcnt3*, *mgat5*, *mgat5b* were all obviously up-regulated, almost all the transcription levels of the glycogenes had significant difference. These results supported the transcriptional profiling from the PCR Array.

### *K*-mean clustering and GO analysis

**Figure 3:**
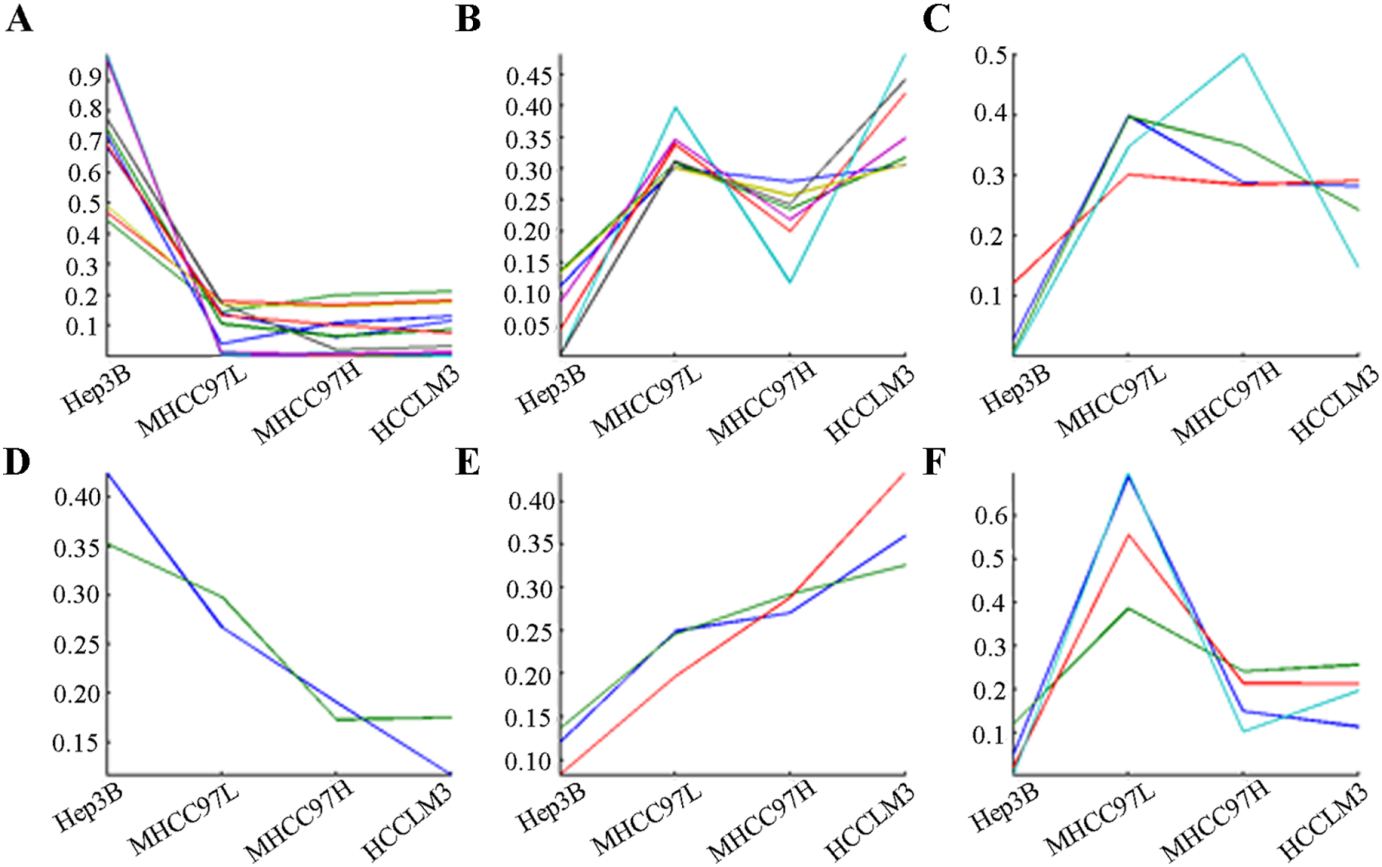
*K-*means clustering of differentially transcribed glycogenes. Each line represented one differentially transcribed glycogene, the shape of lines displayed the expression trend of the differentially transcribed glycogenes in the no-metastatic HCC cell line Hep3B and the HCC cell lines with different metastatic potentials (MHCC97L, MHCC97H and HCCLM3).

**Figure 4:**
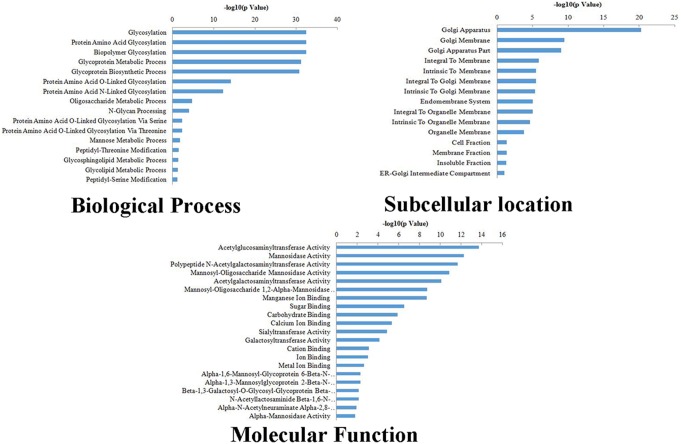
GO analysis of differentially transcribed glycogenes. The differentially transcribed glycogenes were described in 3 categories: biological process, subcellular location, molecular function. GO teams were used to describe three as their attributes of the differentially transcribed glycogenes.

The thirty-three differentially transcribed glycogenes were classified in six clusters according to their trends by *K*-means clustering (Figure 3, Table S2). Among these, only *b4galt5*, *galnt7*, *galnt11* were up-regulated gradually while *man2a2*, *mgat1* were down-regulated step by step, which implied that changes of diverse glycogenes transcription had little related with the level of metastatic potentials. GO teams were used to describe three attributes of the differentially transcribed glycogenes (Figure 4). The differentially transcribed glycogenes were closely related with some types of biological process, such as protein amino acid glycosylation, glycoprotein metabolic process, glycoprotein biosynthetic process. The subcellular distributions of these glycogenes were enriched in golgi apparatus, golgi membrane, and so on. They were associated with molecular functions, like acetylglucosaminyltransferase activity, mannosidase activity, polypeptide N-acetylgalactosaminyltransferase activity.

### Regulatory network by IPA

**Figure 5:**
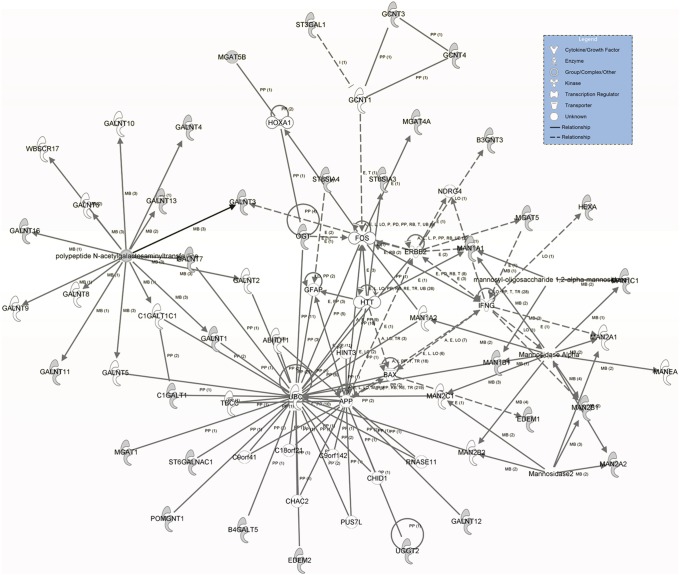
Regulatory network of glycogenes by IPA. The network of glycogenes was derived from the thirty-three differentially transcribed glycogenes. The grey ones represented they were included in the differentially transcribed glycogenes whereas the white ones represented they were not. There were six types of relationship in the network, A: activation, E: expression, M: modification, PD: protein-DNA interaction, PP: protein-protein interaction, T: transcription. Full lines meant a direct action between two nodes, while the dotted lines meant an indirect relationship between two nodes.

All of the thirty-three differentially transcribed glycogenes were uploaded to the database to analyze upstream regulatory events and build the possible regulatory network by the IPA (Figure 5). The molecule type, p-value of overlap and target molecules of the transcription regulators in the regulatory network were described and listed in Table 4. We found that a transcription regulator may control more than one glycogene, such as SIRT1 which can act on *edem1* and *mgat1*. At the same time, it was not difficult to detect that a glycogene may be simultaneously affected by two or more transcription regulators. Instances were described as following: *galnt11* was direct regulated by CLOCK, meanwhile it was indirectly regulated by CCND1. Moreover, *edem1* was directly influenced by XBP1, KCNIP3 and SIRT1, in the meantime, it was indirectly influenced by TMB1M6, IDH1, BAK1. As expected, results showed that the different transcription regulators tended to have different degree of regulation on glycogene expressions and these influences could be measured by the p-value of overlap. The smaller p-value of overlap there was, the greater influence of the regulation there was. For a given glycogene, which of the transcription regulator(s) had more influence could be discriminated by the p-value of overlap. Like *edem1* as a glycogene was influenced by XBP1, SIRT1, TMBIM6, IDH1, BAK1, KCNIP3, nevertheless, KCNIP3 and TMBIM6 play much more important roles. For the seventeen glycogenes in the regulatory network, NCF4, SDC1, SIRT1, CLOCK, TMBIM6 had smaller p-values and it suggested that they may play more important roles in the transcriptional regulation of the differentially transcribed glycogenes.

**Table 4:** Upstream regulators and target molecules in the regulatory network.

Upstream Regulator	EntrezID	Molecule Type	p-value of overlap	Target molecules in dataset
ACSL5	51703	enzyme	0.0246	*galnt13*
ADCYAP1	116	other	0.0467	*galnt7*, *man1a1*
AIRE	326	transcription regulator	0.0023	*mgat1*, *mgat5*
BAK1	578	other	0.0416	*edem1*
CCND1	595	other	0.0418	*galnt11*, *st6galnac1*
CLOCK	9575	transcription regulator	0.0186	*galnt11*, *pomgnt1*
ETS1	2113	transcription regulator	0.0352	*b4galt5*, *mgat5*
ETV4	2118	transcription regulator	0.0416	*b4galt5*
HIST1H1A	3024	other	0.0003	*b4galt5*, *man2b1*, *ogt*
HIST1H1T	3010	other	0.0003	*b4galt5*, *man2b1*, *ogt*
IDH1	3417	enzyme	0.0416	*edem1*
KCNIP3	30818	transcription regulator	0.0232	*edem1*
NCF4	4689	enzyme	0.0146	*st3gal1*
SDC1	6382	enzyme	0.0117	*galnt3*
SIRT1	23411	transcription regulator	0.0125	*edem1*, *mgat1*
STAT5B	6777	transcription regulator	0.0229	*hexa*, *st8sia4*
TFEB	7942	transcription regulator	0.0445	*hexa*
TMBIM6	7009	other	0.0189	*edem1*
XBP1	7494	transcription regulator	0.0340	*edem1*, *edem2*

### Validation in clinical cancer specimens by qRT-PCR

**Figure 6:**
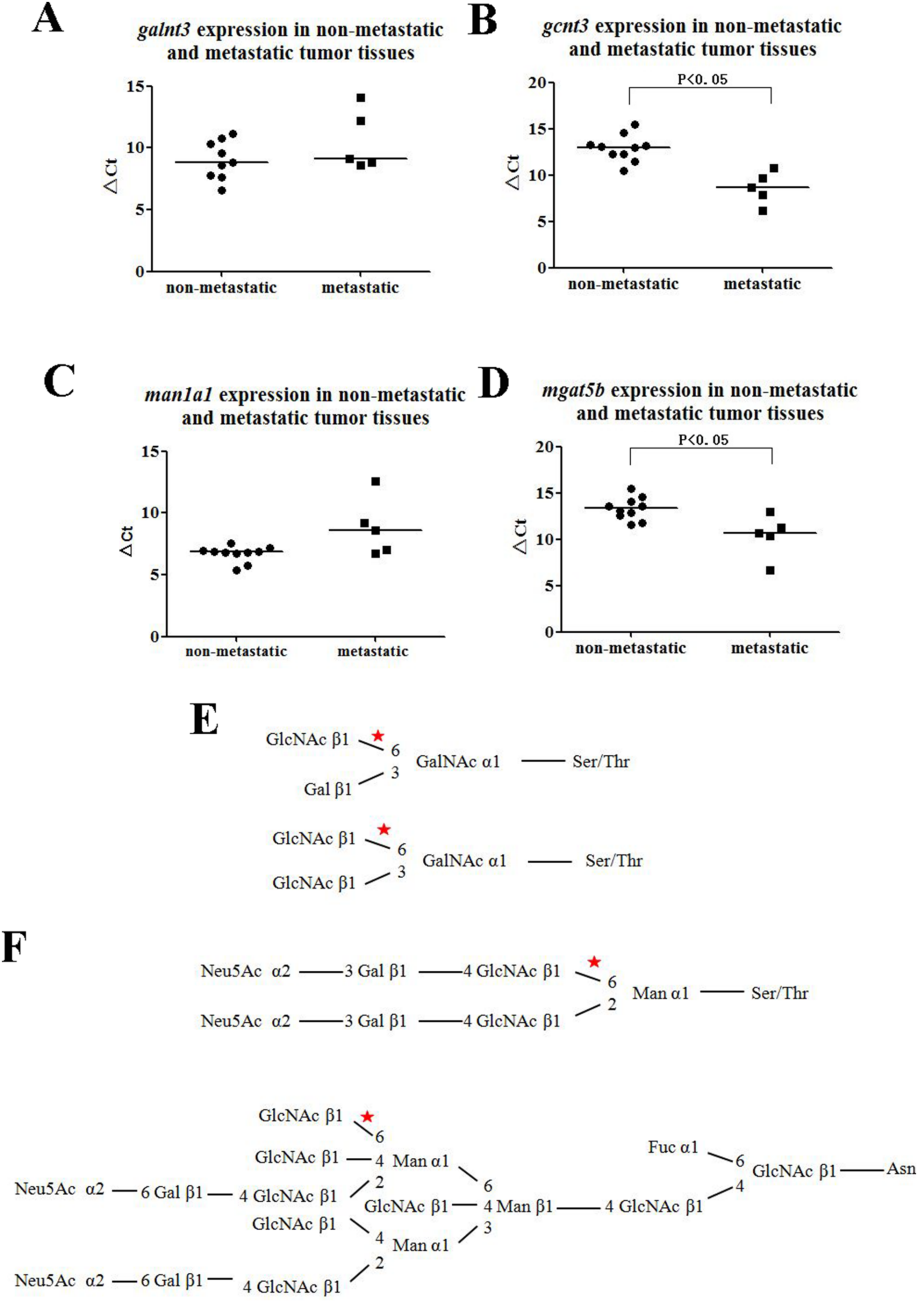
Validation in non-metastatic and metastatic tissue samples by qRT-PCR. A, B, C, D were the transcription levels of *galnt3*, *gcnt3*, *man1a1*, *mgat5b*, respectively. The housekeeping gene, *β-Actin* was used to normalize the expression levels in the subsequent quantitative analyses, and the higher value of ΔCt of the glycogene, the lower its transcription level. E, *gcnt3* involved in the glycan biosynthetic pathways. F, *mgat5b was* correlated with the pathway of glycan biosynthetic.

Four differentially transcribed glycogenes, including *galnt3*, *gcnt3*, *man1a1*, *mgat5b* were selected to detect the expressions in clinical cancer specimens and their results were shown in Figure 6. Although the transcription levels of *galnt3* and *man1a1* in metastatic HCC clinical cancer specimens were lower than in the non-metastatic ones, there were no significant differences (P = 0.246, P = 0.108, respectively). While the divergent trends of *gcnt3* and *mgat5b* between non-metastatic and metastatic HCC clinical cancer specimens were statistically significant (P = 0.002, P = 0.04, respectively) and they were all up-regulated in metastatic HCC clinical cancer specimens, which trends were as same as in human HCC cell lines and their orthotopic xenograft tumors. *gcnt3* (glucosaminyl (N-acetyl) transferase 3, mucin type) is required to form GlcNAcβ1→6Gal/GalNAc (Figure 6E) [26] and *mgat5b* (mannosyl (α-1,6-)-glycoprotein β-1,6-N-acetyl-glucosaminyltransferase, isozyme B) acts on the GlcNAc β1,2-Manα1-Ser/Thr moiety, forms a 2,6-branched structure in brain O-mannosyl glycan (Figure 6F) [27].

## Discussion

A growing number of researches indicated that the structural modifications of cell membrane glycans had correlation with disease progression, metastasis, and poor prognosis [28], [29]. Essentially, cancer could be regarded as a molecular disease of cell membrane glycans because cancer cells were characterized by aberrant glycosylation of the surface membrane [30]. Abnormal glycosylation was also highly associated with HCC progression. The alterations of glycan branched structures of glycoproteins, such as GlcNAc-branched N–glycans, sialic acid and core fucosylation, were demonstrated to be highly expressed in HCC and these changes were closely related to the transcriptional and translational levels of the glycogenes [31]–[34]. It was generally known that AFP-L3, which was the LCA-bound fraction of AFP, as a new generation of tumor marker for HCC had been widely used in clinic. It had been reported that malignant liver cells produce AFP-L3, even when HCC was at its early stage [34]. In the meantime, *fut8* was known to be a key enzyme of fucosylation and it had been implicated in the production of AFP-L3 [35], [36]. This example suggested that some abnormal glycosylation and the differentially transcribed glycogenes might be potential markers of biologic malignancy of HCC and maybe they could be used for identifying subtypes of HCC and elevating the possibility of individual treatment, hence the further research was needed.

In current study, we obtained and validated the transcriptional profiling of glycogenes. This was the first study to select the transcriptional profiling of glycogenes associated with HCC metastasis by PCR Array and it was a comprehensive analysis of glycogenes with relevant N-linked glycosylation, O-Linked glycosylation, glycosphingolipids in human HCC cell lines, orthotopic xenograft tumors and clinical cancer specimens.

The metastatic potential of HCC cell lines in our experiments were strengthening step by step. Hep3B, the non-metastatic cell line from ATCC, was established from a patient of 8-year-old black boy. Nevertheless, MHCC97L, MHCC97H and HCCLM3, all of them can metastasize, whose metastatic potential were increasing in general [35]. The original source of these cell lines was MHCC97 which derived from the LCI20 tumor line (male HCC) and their lung metastatic rate were 40%, 100% and 100%, respectively. The node rate of HCCLM3 was 60% [36]. We found thirty-three differentially transcribed glycogenes by the Human Glycosylation RT^2^ Profiler PCR Array with differences greater than 2-fold (P≤0.05) as a criteria. A glycomics study using glycogene microarray which contained 115 genes by Kang et.al reported eighteen glycogenes were up-regulated in high metastatic potential HCCLM3 cell lines in comparison to Hep3B cell lines, while eleven glycogenes were down-regulated [4]. Guo et.al’s study using qRT-PCR for quantification found that six glycogenes *b3galt1*, *fut8*, *gal3st2*, *mgat5*, *st3gal5 and st6gal1* were overexpressed in MHCC97H cell lines compared with those in MHCC97L cell lines; conversely, five glycogenes, *b3gnt6*, *b3gnt7*, *galnt6*, *mgat3 and st8sia2* were down-regulated [7]. However, Kang et.al only compared the high metastatic potential HCC cell lines HCCLM3 to non-metastasis cell lines Hep3B and Guo et.al only compared two metastasis cell lines (MHCC97L and MHCC97H). Comparisons both of them carried out were between two cell lines, while our results were more confident than theirs with four cell lines to be compared, and all of the differently transcribed glycogenes had been identificated in orthotopic xenograft tumors and validated in clinical cancer specimens. Kang et.al used glycogene microarray, by contrast, we used the PCR-Array. It was cheaper than by gene microarrays and its experimental conditions could be easily controlled [37]. In contrast with Guo et.al’s result by qRT-PCR, our findings obtained more differentially transcribed genes. Additionally, apart from the glycogenes which have been reported previously like *mgat1*, *mgat5 and st3gal1*, some glycogenes such as “UDP-N-acetyl-α-D-galactosamine: polypeptide N-acetylgalactosaminyltransferase” family members: *galnt3*, *galnt4*, *galnt11*, *galnt13*, and “mannosidase, α class” family members, for instance, *man1b1*, *man1c1*, *man2a2*, their different expressions were associated with HCC metastasis are the first reported. This research revealed that glycogenes which could generate and alter sialylated glycan structures or β-1, 6-GlcNAc branched N-Glycan were significantly up-regulated in the metastatic HCC cell lines and their orthotopic xenograft tumors. Sialic acids could prevent cell-cell interactions through charge repulsion effects [38]. Tumor cell lines with more invasive ability expressed more heavily sialylated glycan structures. *mgat5* could advance tumor growth and metastasis by destructing the extracellular matrix or boost the production of angiogenic factors by a metal ion-dependent serine protease. *mgat5* could also promote tumor metastasis by catalyzing sugar chain which can change the function of angiogenic factors or by directly increasing the gene transcription of angiogenic factors [39].

The qRT-PCR analysis of the transcribed glycogenes in the human HCC cell lines and their orthotopic xenograft tumors provided evidences of the same changing trends in statistics (P<0.05) to support the analysis of PCR-array results. Nevertheless, we found that the respective fold-change value of comparison results within metastatic HCC cell lines and their orthotopic xenograft tumors had no obvious gradually change trend. It revealed that the transcriptional of glycogenes was highly related with metastasis no matter whether their metastatic potentials were high or low. Therefore, we suggested that our future studies about glycosyltransferases should more focus on comparison of whether these cells or orthotopic xenograft tumors were able to metastatic or not rather than the difference of their metastatic potentials.

The regulatory network of part of the differentially transcribed glycogenes was obtained by the IPA, and it included six types of relationship and nineteen upstream regulators. Aside from regulators, current data suggests that miRNA is related with the expressions of glycogenes. Human and rodents HCC cell lines were transfected with miR-122 and miR-34a and the result demonstrated that forced expression of these miRNAs was able to induce a decrease of *fut8* levels and also to affect core fucosylation of secreted proteins [40]. The passenger strand miR-17-3p repressed expression of *galnt7*, which regulates metabolism of liver toxin galactosamine [41]. Further analysis including glycogenes regulation may elucidate the changes of the expressions of glycogenes.

The validation in clinical cancer specimens showed some differences of the transcription levels of glycogenes in clinical tissue samples were not significant. It might be caused by the part of the differences among cell lines, orthotopic xenograft tumors and clinical tissue samples themselves. But it was important to note there were still a substantial part of glycogenes which were differentially transcribed between non-metastatic and metastatic tissues. This evidence suggested there might be some potential novel drug targets and biological markers to predict metastasis of HCC in the differentially transcribed genes. To validate the transcription levels of the remaining differentially transcribed glycogenes, further investigation is needed and this part of the work is undergoing. Moreover, it could not be neglected that our study is the first time to provide evidences in human HCC cell lines, orthotopic xenograft tumors and clinical tissue samples that the up-regulated of *gcnt3* and *mgat5b* were associated with HCC metastasis.

In summary, this study identified thirty-three differentially transcribed glycogenes by the Human Glycosylation RT^2^ Profiler PCR Array. A total of seven glycogenes, including *c1galt1*, *galnt3*, *gcnt3*, *man1a1*, *mgat4b*, *mgat5*, *mgat5b*, were further identified by qRT-PCR in HCC cell lines and their orthotopic xenograft tumors. All of these differentially transcribed glycogenes were analyzed by *K*-means and GO analysis, and were uploaded to the IPA database for regulatory network analysis. What was more, *galnt3*, *gcnt3*, *man1a1* and *mgat5b* were selected to further validation in clinical cancer specimens and this is the first report that the up-regulated of *gcnt3* and *mgat5b* were associated with HCC metastasis. The differentially transcribed glycogenes could be the potential targets for the novel drugs and might act as the potential biological markers to predict metastasis of HCC.

## Supporting Information

Table S1 **General information of non-metastatic and metastatic HCC patients.**
(DOCX)

Table S2 **The K-means class of the differentially expression glycogenes.**
(DOCX)
