# Supplementary material for: The Transcriptional Profiling of Glycogenes Associated with Hepatocellular Carcinoma Metastasis
Source: PLoS One. 2014 Sep 18;9(9):e107941. doi: 10.1371/journal.pone.0107941 (PMC4169445; doi:10.1371/journal.pone.0107941)
Supplement: Table S1 — General information of non-metastatic and metastatic HCC patients. (DOCX) [file pone.0107941.s001.docx]

Table S1 General information of non-metastatic and metastatic HCC patients.

|  |  |  | **non-metastatic** | **metastatic** |
| --- | --- | --- | --- | --- |
| Number of individuals | | | 10 | 5 |
| Gender(male/female) | | | 6(60%)/4(40%) | 4(80%)/1(20%) |
| Age(years) | | | 65±10.65 | 53±16 |
|  |  |  | （mean±SD） | （mean±SD） |
| AFP(ng/mL) | | | 2382±5189 | 1311±2279 |
|  |  |  | （mean±SD） | （mean±SD） |
| BCLC | | | A1(n=2), A2(n=3), A4(n=3), B(n=2) | A3(n=1), B(n=2), C(n=2) |
