# Supplementary material for: The Transcriptional Profiling of Glycogenes Associated with Hepatocellular Carcinoma Metastasis
Source: PLoS One. 2014 Sep 18;9(9):e107941. doi: 10.1371/journal.pone.0107941 (PMC4169445; doi:10.1371/journal.pone.0107941)
Supplement: Table S2 — The K-means class of the differentially expression glycogenes. (DOCX) [file pone.0107941.s002.docx]

Table S2 the *K*-means class of the differentially expression glycogenes.

| **glycogene** | ***K*-means class** |
| --- | --- |
| *a4gnt* | 1 |
| *b3gnt3* | 3 |
| *b4galt5* | 5 |
| *c1galt1* | 1 |
| *edem1* | 6 |
| *edem2* | 2 |
| *galnt1* | 1 |
| *galnt11* | 5 |
| *galnt12* | 1 |
| *galnt13* | 1 |
| *galnt3* | 1 |
| *galnt4* | 6 |
| *galnt7* | 5 |
| *galntl1* | 1 |
| *gcnt3* | 3 |
| *gcnt4* | 1 |
| *hexa* | 3 |
| *man1a1* | 1 |
| *man1b1* | 2 |
| *man1c1* | 6 |
| *man2a2* | 4 |
| *man2b1* | 1 |
| *mgat1* | 4 |
| *mgat4a* | 1 |
| *mgat5* | 2 |
| *mgat5b* | 2 |
| *ogt* | 2 |
| *pomgnt1* | 2 |
| *st3gal1* | 2 |
| *st6galnac1* | 3 |
| *st8sia3* | 1 |
| *st8sia4* | 6 |
| *uggt2* | 1 |
